# The effects of cinnamaldehyde and eugenol on human adipose-derived mesenchymal stem cells viability, growth and differentiation: a cheminformatics and *in vitro* study

**Published:** 2016

**Authors:** Abdorrahim Absalan, Seyed Alireza Mesbah-Namin, Taki Tiraihi, Taher Taheri

**Affiliations:** 1 *Department of Clinical Biochemistry, Faculty of Medical Sciences, Tarbiat Modares University, Tehran, Iran*; 2 *Department of Anatomical Sciences, Faculty of Medical Sciences, Tarbiat Modares University, Tehran, Iran*; 3 *Shefa Neuroscience Research Center, Khatam Alanbia Hospital, Tehran, Iran*

**Keywords:** *Stem cell*, *Cell viability*, *Quantitative structure-activity Relationship*, *Cinnamaldehyde*, *Eugenol*

## Abstract

**Objective:**

The aim of this study was to estimate the cheminformatics and qualitative structure-activity relationship (QSAR) of cinnamaldehyde and eugenol. The effects of cinnamaldehyde and eugenol on the viability, doubling time and adipogenic or osteogenic differentiations of human adipose-derived mesenchymal stem cells (hASCs) were also investigated.

**Materials and Methods:**

QSAR and toxicity indices of cinnamaldehyde and eugenol were evaluated using cheminformatics tools including Toxtree and Toxicity Estimation Software Tool (T.E.S.T) and molinspiration server. Besides, their effects on the hASCs viability, doubling time and differentiation to adipogenic or osteogenic lineages were evaluated.

**Results:**

Cinnamaldehyde is predicted to be more lipophilic and less toxic than eugenol. Both phytochemicals may be developmental toxicants. They probably undergo hydroxylation and epoxidation reactions by cytochrome-P450. The 2.5 µM/ml cinnamaldehyde and 0.1 µg/ml eugenol did not influence hASCs viability following 72 hr of treatment. But higher concentrations of these phytochemicals insignificantly increased hASCs doubling time till 96 hr, except 1 µg/ml eugenol for which the increase was significant. Only low concentrations of both phytochemicals were tested for their effects on the hASCs differentiation. The 2.5 µM/ml cinnamaldehyde and 0.1 µg/ml eugenol enhanced the osteogenesis and decreased the adipogenesis of hASCs meaningfully.

**Conclusion:**

According to the cheminformatics analysis and *in vitro* study, cinnamaldehyde and eugenol are biocompatible and low toxic for hASCs. Both phytochemicals may be suitable for regenerative medicine and tissue engineering when used at low concentrations, but maybe useful for neoplastic growth inhibition when used at high concentrations.

## Introduction

Human adipose-derived stem cells represent a proper source for stem cell therapy. They may be useful in regenerative medicine (Gimble et al., 2007 ▶). Phytochemical compounds may target various signal transduction proteins and change the cell fate (Alarcón de la Lastra and Villegas, 2005 ▶; Ho et al., 2010 ▶). Such targeting could exert ageing or anti-ageing effects on proliferating stem cells. Also, researchers have reported anti-ageing and antioxidant characteristics for herbal ingredients (Cai et al., 2004 ▶; Wong et al., 2006 ▶). On the other hand, direct binding to signal transduction molecules has been reported as a mechanism of aging induction. Green tea and turmeric ingredients are examples of this matter (Aggarwal et al., 2006 ▶; Kuzuhara et al., 2008 ▶). Both aging and antiaging effects of herbal ingredients are notable when considered for targeting cancer and stem cells, respectively. Aging or antiaging properties of phytochemicals may belong to the toxic or anti-oxidant properties of their ingredients. In this regard, the current study has evaluated cheminformatics estimation of cinnamaldehyde and eugenol toxicity. Also, the effect of cinnamaldehyde and eugenol on the viability, doubling time and morphologic differentiation of human adipose-derived stem cells (hASCs) were studied.

## Materials and Methods

**Estimating toxicity and qualitative structure-activity relationship (QSAR)**

According to the Lipinski rule of five (RO5), a chemical compound could be considered as a drug with good absorption and permeation through cell membranes if it has five features: 1. Its H-bond donor atoms do not exceed more than five; 2. Its molecular mass is less than 500 Dalton; 3. The number of rotatable bonds is than or equals ten bonds; 4. The partition coefficient (Log P) of its solubility in octanol to water phases is less than five; 5. It has not more than 10 nitrogen and oxygen atoms in its structure (Lipinski et al., 2012 ▶). In the present study, molinspiration server (http://www.molinspiration.com) was used to address RO5 of cinnamaldehyde and eugenol. Three dimensional cheminformatics structure of cinnamaldehyde and eugenol were downloaded in mol2 or SMILES format, and from ZINC online database (http://zinc.docking.org) (Irwin and Shoichet, 2005). Such 3D structures were necessary for cheminformatics and toxicity virtual analysis. Toxtree software (http://toxtree.sourceforge.net/) and toxicity estimation software tool (T.E.S.T) (http://www.epa.gov/nrmrl/std/qsar/qsar.html) were also used to evaluate qualitative structure-activity relationship (QSAR) for cinnamaldehyde and eugenol. These software packages estimate the probable lethal doses or concentrations of chemicals for some creatures, cinnamaldehyde and eugenol biodegradability, genotoxicity, nongenotoxic carcinogenicity, DNA and protein binding, cytochrome-P450 catabolism end products, bioaccumulation, and developmental toxicity or mutagenicity features. 

**Adipose-derived mesenchymal stem cells (ASCs) isolation and evaluation of cell determinants (CD)**

Human ASCs (hASCs) were separated from adipose tissue of a 34 year old pregnant woman, during caesarean section and after fulfilling consent form. Briefly, dissected adipose tissue was drained using phosphate buffer, digested with collagenase I, and pelleted with centrifugation and then the pellet was seeded in Dulbecco's Modified Eagle Medium (DMEM) (Invitrogen) plus fetal bovine serum (FBS). Stem cells attached to the flasks after 7-10 days later the seeding. The cells were kept in DMEM + 0.1% antibiotics + 10% FBS up to reach 90% confluence, in a humidified incubator with 5% CO_2_. hASCs were fixed with 4% paraformaldehyde (Sigma-Aldrich), treated with 1:300 diluted primary antibodies overnight, then treated for 1 hr with 1:500 dilution of secondary antibodies, and for a few seconds with propidium iodide (Sigma-Aldrich) as counter stain. Primary antibodies were from Abcam Company and used to show that dividing adipose-derived cells are mesenchymal stem cells. Anti-human CD45 and CD56 antibodies were used as human mesenchymal stem cells (hMSCs) negative CD markers. Anti-human CD73, CD90 and CD105 were used as the positive markers for hMSCs. Secondary antibodies were conjugated to fluorescein isothiocyanate (FITC) (Millipore). 

**Cell viability assay**

Cinnamaldehyde, eugenol and dimethyl sulfoxide (DMSO) were from Sigma-Aldrich. Here, 250 µl of a cell suspension, with 2500 cells/ml in DMEM+10% FBS+0.1% penicillin/streptomycin, was added to the wells of a 96-well plate. They included eight different suspensions: 1. Control group had no additive; 2. DMSO group containing 0.01% DMSO as solvent control; 3. Cinnamaldehyde 2.5 µM/ml plus 0.01% DMSO; 4. Cinnamaldehyde 5 µM/ml plus 0.01% DMSO; 5. Cinnamaldehyde 7.5 µM/ml plus 0.01% DMSO µM/mL; 6. Eugenol 0.1 µg/ml plus 0.01% DMSO; 7. Eugenol 0.5 µg/ml plus 0.01% DMSO; 8. Eugenol 1 µg/ml plus 0.01% DMSO. Culture plates were kept for 24, 48 and 72 hr and cell viability was assessed for control and treatment groups according to the Cell Titer 96^®^ AQueous Assay kit which contains the 3-(4,5-dimethylthiazol-2-yl)-5-(3-carboxymethoxyphenyl)-2-(4-sulfophenyl)-2H-tetrazolium (MTS) reagent (Promega).

Optical density (OD) of each well was measured at 490 nm wavelength and viability percent chart was plotted using Excel software. Each group was evaluated in triplicate repeats. ODs of eight mentioned groups were analyzed statistically by one way ANOVA and Games-Howell test with 95% confidence interval (CI) to compare the cell viability among studied groups or double comparisons, respectively.

**Doubling time assessment**

Eight different suspensions were prepared in primary culture media as described for cell viability assay. Exactly 800 µl of each of the above-described cell suspensions were added to 24-well plates. After 24, 48, 72 and 96 hr, the cells were detached using 0.25% trypsin-EDTA solution (GIBCO). Viable and dead cell counting was done using 0.4% trypan blue solution and with a hemocytometer slide. Doubling time was analyzed and curves were plotted using an online doubling time calculator at the URL http://www.doubling-time.com/compute.php. Each group was tested in quadruplicates. Statistical analysis was done using ANOVA and Tukey-HSD with 95% CI to compare the variance of doubling times among all or between two groups, respectively.

**Adipogenic and osteogenic differentiation**

According to the doubling time result of present research, four groups of above-mentioned hASCs which had the minimum doubling time, were selected for studying the effect of cinnamaldehyde and eugenol on the differentiation of hASCs to adipocyte and osteocyte. The adipogenic and osteogenic differentiation was evaluated in hASCs in untreated, 0.01% DMSO-treated, 2.5 µM/ml cinnamaldehyde plus 0.01% DMSO and 0.1 µg/ml eugenol plus 0.01% DMSO groups. Differentiation was performed according to the previously described method (Bunnell et al., 2008 ▶) to assess cinnamaldehyde or eugenol effects on the morphological differentiation of hASCs. Briefly, hASCs were seeded in the DMEM with 10% FBS plus antibiotics. The culture media was changed every other day. After the cells reached up to 70 to 80 percent confluence, differentiation medium was added. All chemicals were purchased from Sigma-Aldrich. 

The adipogenic media ingredients included bovine insulin 0.115 mg/dl, dexamethasone 0.4 mg/dl, rosiglitazone 180 µg/dl, D-Pantothenics acid 0.75 mg/dl, 3-Isobutyl-1-methylxanthine 5.6 mg/dl, biotin 1.617 mg/dl in DMEM with 3% FBS, with any additive for control group, with 0.01 DMSO, with 2.5 µM/ml cinnamaldehyde and 0.01% DMSO or 0.1 µg/ml eugenol and 0.01% DMSO. DMSO was the solvent of cinnamaldehyde and eugenol. Oil red staining was done on day 16 of differentiation. 

The osteogenic media ingredients include: Sodium-2 phosphate L-ascorbat 0.005 g/dl, beta-glycerol phosphate 0.216 g/dl, dexamethasone 0.4 mg/dl in DMEM with 10% FBS, with no additive for control group, 0.01 DMSO, 2.5 µM/ml cinnamaldehyde plus 0.01% DMSO or 0.1 µg/ml eugenol plus 0.01% DMSO. Alizarin red staining was done on day 19 of differentiation. 

We examined differentiated cells microscopically. Alizarin red stains the calcium deposits of osteocytes. Oil red stains the adipocytes cytoplasm, then the fat vacuoles became more visible and detectable. For semi-quantification of differentiation rate in adipocytes and osteocytes ImageJ and TotalLab TL120 software were used, respectively. For each untreated and treated groups, at least 5 images were taken and analyzed by the mentioned software. The ImageJ calculated the percentage of red color of calcium deposits which were stained with Alizarin red. The TotalLab TL120 calculated the fat vacuole counts in the microscopic images. Differentiation semi-quantities were obtained from each software and statistical analysis was done using ANOVA and Tukey-HSD for double comparisons with 95% CI.

## Results

**Lipinski´s RO5, QSAR and toxicity estimations**

Lipinski´s RO5 criteria and calculated toxicity indices of cinnamaldehyde and eugenol are shown in Table 1. Calculated features included 1. Partition coefficient (LogP) where the higher value means the better lipophilicity of chemical; 2. The number of oxygen or nitrogen atoms which are hydrogen acceptor; 3. The hydroxyl or amine group (nOHNH) counts, which are hydrogen donor; 4. The molecular weight where the compounds are more bioavailable with molecular weight less than 500 Dalton; 5. The rotatable bonds (nrotb) counts which is better to be less than or equal to 10 bonds to make the chemical compound more bioavailable (Lipinski et al., 2012 ▶). Other features included topological polar surface area (TPSA) for which, values lesser than or equal to 140 show better membrane permeability for a drug (Veber et al., 2002 ▶). Overall, QSAR estimated features showed that both phytochemicals are of low toxicity. Cinnamaldehyde was not genotoxic, but eugenol was estimated to be potentially carcinogenic or mutagenic, because of its alkenylbenzene group, but none of them was estimated to be non-genotoxic carcinogen. Both phytochemicals may bind to DNA or proteins and were estimated as developmental toxicants. The two phytochemicals were predicted to be easily biodegradable. Cytochrome-P450 could metabolize them through aromatic or aliphatic hydroxylation, epoxidation and dealkylation (Tables 2 and 3). According to half maximal lethal concentration (LC50) for 96 hr exposure time which was obtained from T.E.S.T software, cinnamaldehyde is less lethal than eugenol for fathead minnow (*Pimephales promelas*). But it was estimated to be more lethal for *Daphnia magna* and *Tetrahymena pyriformis* for 48 hr exposure time. According to rat oral LD50, eugenol is more lethal than cinnamaldehyde but their lethal doses were not much different.

**Human ASC’s CD markers**

**Figure 1:**
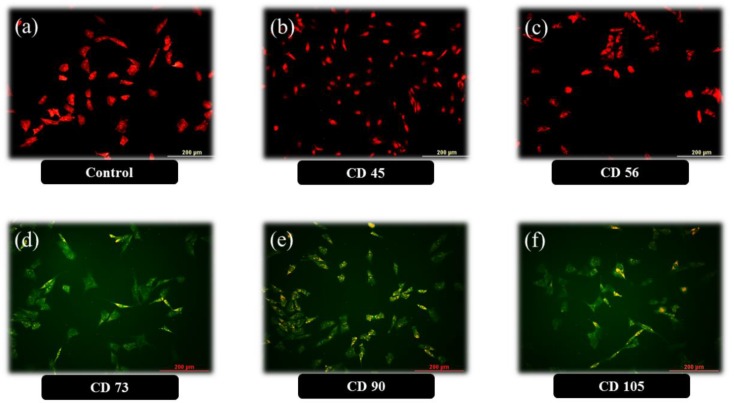
Confirmation of human adipose-derived mesenchymal stem cells isolation using immunocytochemistry. After separation from fat tissue, the cells were seeded in DMEM+10% FBS plus 0.1% antibiotics and kept till reaching 70-80% confluence. Thereafter, growing cells were fixed with 4% paraformaldehyde, treated with primary antibodies overnight, secondary antibodies 1 hr, followed by propidium iodide treatment for a few seconds and washing with phosphate buffer. Control group was not treated with primary antibodies. Florescent microscopy images confirmed that the cells were CD45 and CD56 negative, but CD73, CD90 and CD105 positive. This phenotype confirmed that the isolated cells are human mesenchymal adipose-derived cells (hASCs

For confirmation of hASCs isolation, CD markers were checked (Figure 1). Isolated cells were CD45 and CD56 negative, but CD73, CD90 and CD105 positive. These phenotypes confirmed that isolated cells are adult mesenchymal stem cells (de Villiers et al., 2009 ▶; Gimble and Nuttall, 2011 ▶; Izadpanah et al., 2006 ▶).

**Table 1:** Toxicological features of cinnamaldehyde and eugenol. Lipinski´s RO5 features calculated by the Molinspiration web server. Other toxicity indices were calculated using off-line Toxtree and T.E.S.T software

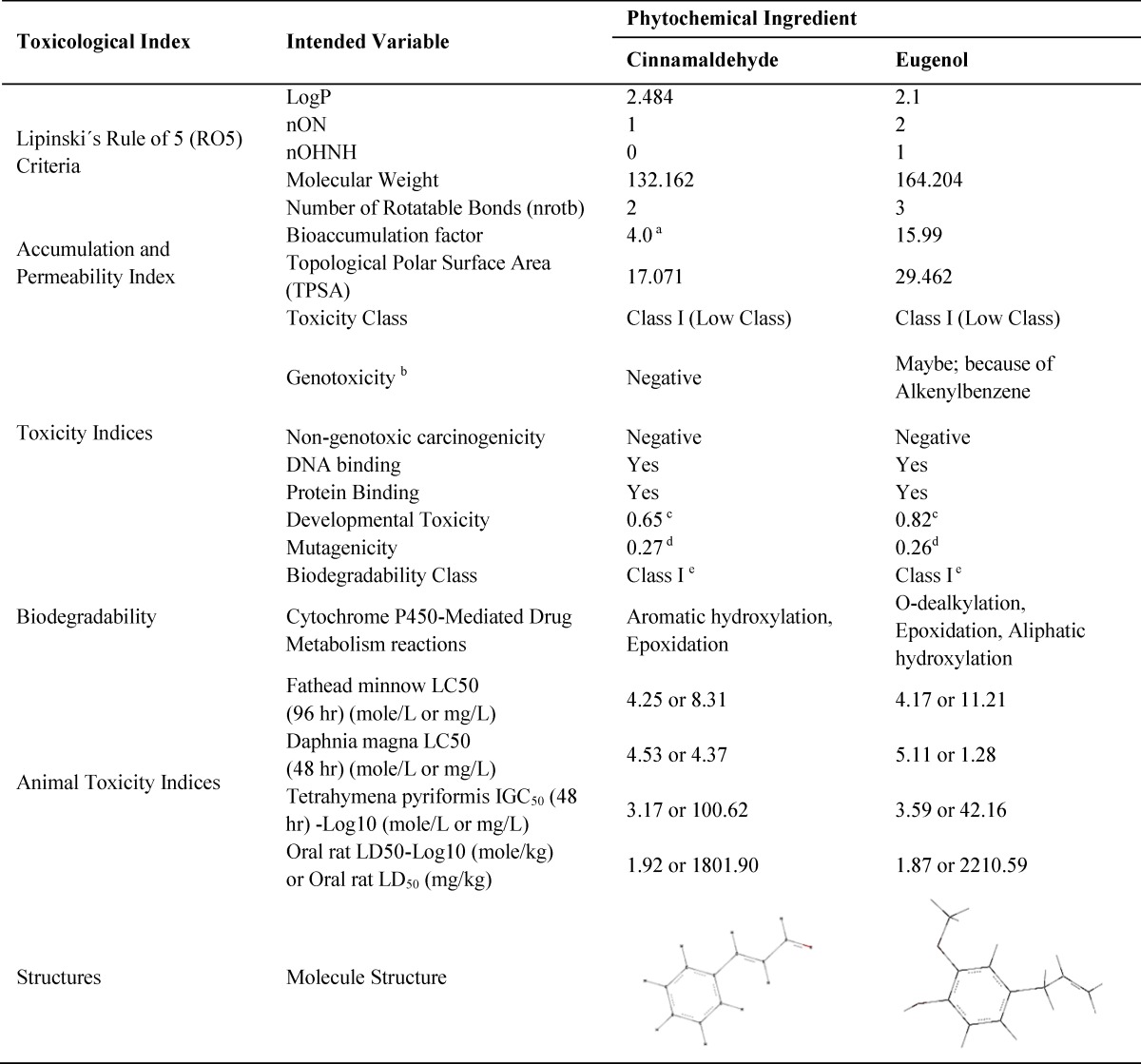

Comments: a calculated for Cinnamyl alcohol form. b Carcinogenicity and mutagenicity. c Developmental toxicant. d Mutagenicity negative. e Easily biodegradable.

**Table 2:** Predicted reactions and end products of cinnamaldehyde after metabolism by cytochrome-P450 using Toxtree software

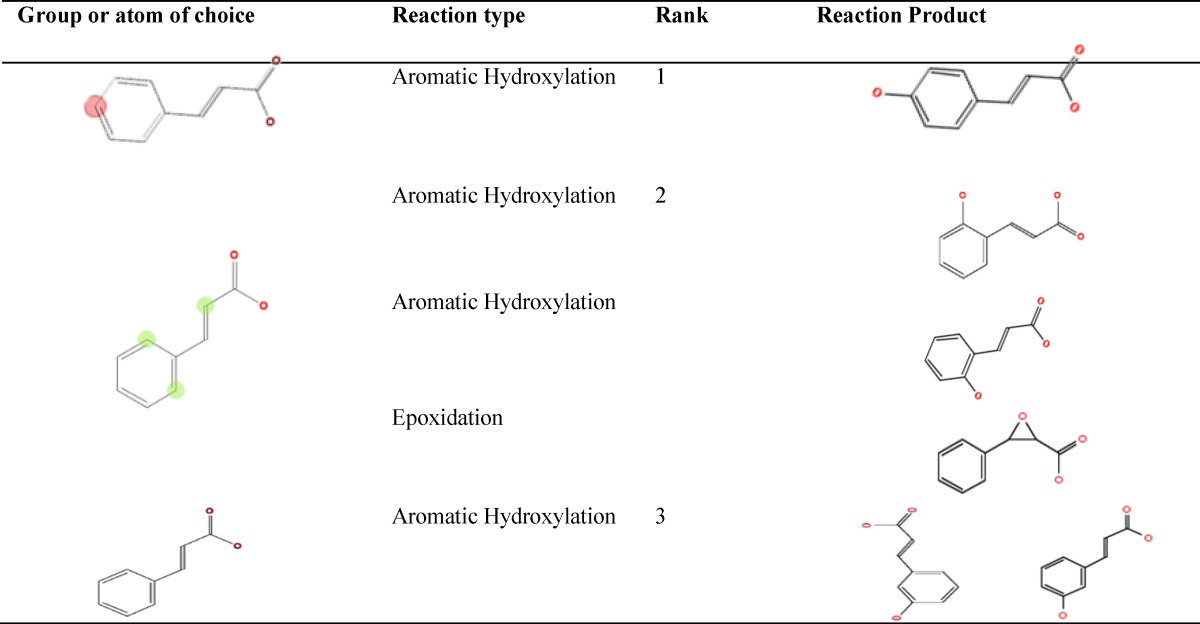

**Table 3:** Predicted reactions and end products of eugenol after metabolism by cytochrom-P450 using Toxtree software.

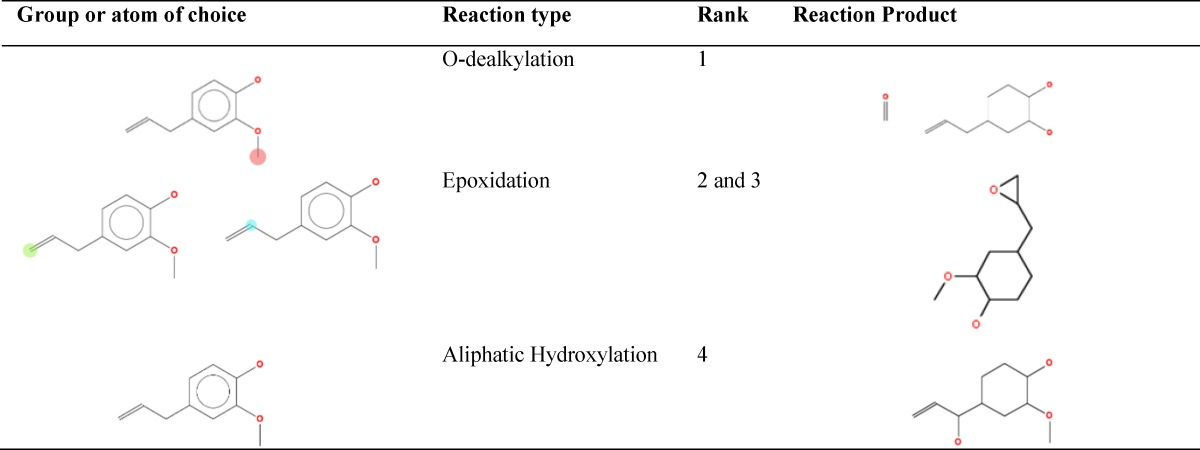

**Cell viability analysis**

**Figure 2:**
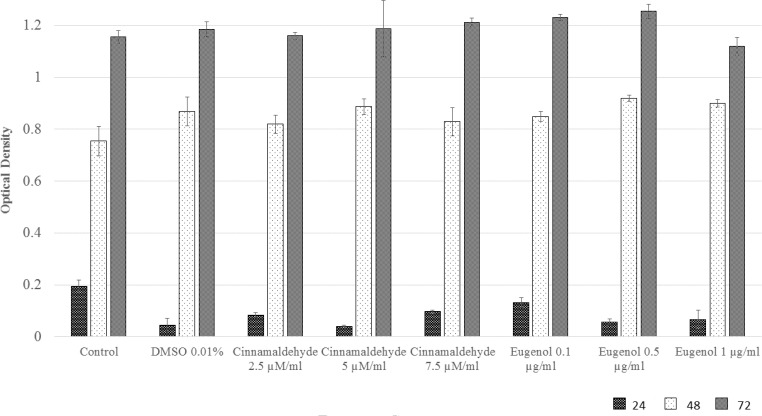
MTS viability test of hASCs under eight different growth status as mentioned under the columns. HASCs were seeded in 96 well culture plates and kept for 24 hr, 48 hr or 72 hr in cell culture standard status. After the mentioned times the OD of each well was measured in 490 wave length exactly after 1 hour incubation with MTS reagent. Although, there is a significant difference between OD of eight groups after 24 hr (P=0.001) but not for 48 hr (P=0.149) or 72 hr (P=0.500) treatment

Figure 2 shows that there was a significant OD difference among eight groups after 24 hr (p=0.000). But there were no difference 48 (p=0.149) or 72 hr (p=0.500) after treatment.

**Doubling time analysis**

**Figure 3.**
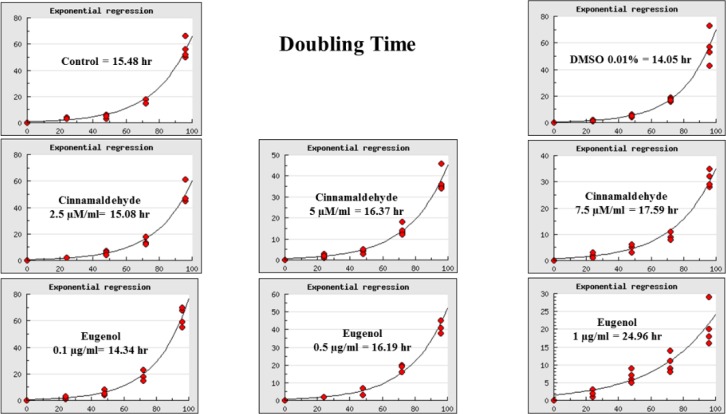
Growth curve and doubling time analysis. Eight suspensions of hASCs were analyzed for doubling time as described in the Material and Methods section. The lowest concentrations of cinnamaldehyde (2.5 µM/ml) or eugenol (0.1 µg/ml) did not significantly change the doubling time compared to control and 0.01% DMSO-treated hASCs (CI=0.95; p>0.05). However, the higher concentrations increased the time of duplication in a concentration-dependent way. Only 1 µg/ml eugenol increased the doubling time of hASCs meaningfully compared to all other seven groups (p= 0.000

**Figure 4:**
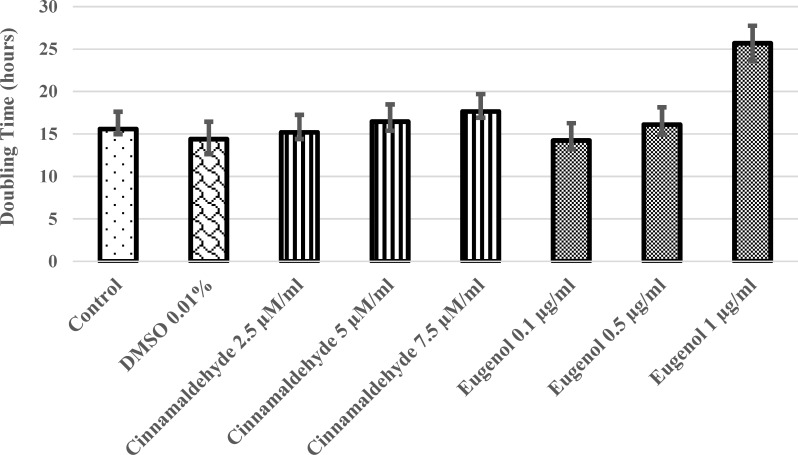
The doubling time chart. The doubling time is not significantly increased in treatment categories compared to control or 0.01% DMSO-treated (p> 0.05), except 1 µg/ml eugenol-treated hASCs (p= 0.000). An increased concentration-dependent doubling time was obvious for both cinnamaldehyde and eugenol-treated hASCs

After 24, 48, 72 and 96 hours, the cells detached from culture dishes using trypsin-EDTA solution. As evaluated by trypan blue exclusion method, studied groups had significantly different doubling times (p=0.000 for ANOVA). However, there were a significant difference among 7.5 µM/ml cinnamaldehyde or 1 µg/ml eugenol as compared to control and 0.01% DMSO-treated cells (p< 0.05 for Tukey-HSD). Cinnamaldehyde and eugenol increase the doubling time of hASCs in a concentration-dependent way (Figures 3 and 4). However, only 1 µg/ml concentration of eugenol significantly changed the doubling time of hASCs as compared to all other 7 groups (p= 0.000). As the purpose of this study was to find a proper concentration of cinnamaldehyde and eugenol with low toxicity on the hASCs, 2 µM/ml of cinnamaldehyde and 0.1 µg/ml of eugenol were selected according to cheminformatics, cell viability and doubling time analysis. These low concentrations were predicted to have the lowest toxicity on the hASCs. The selected concentrations were used to assess the effect of cinnamaldehyde and eugenol on the differentiation of hASCs to adipocytes and osteocytes. Detailed method of surveying differentiation was described in Materials and Methods section.

**Adipogenic and osteogenic differentiation**

**Figure 5:**
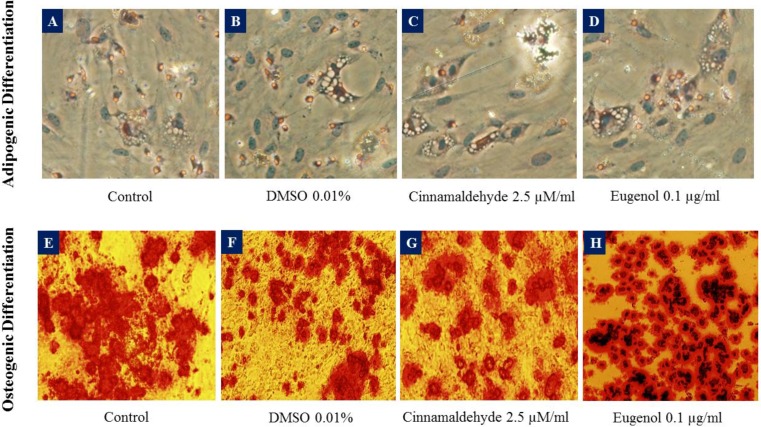
Sample images of adipogenic (A-D) and osteogenic (E-H) differentiation of human adipose-derived stem cells in untreated control, 0.01% DMSO-treated, 2.5 µM/ml cinnamaldehyde-treated and 0.1 µg/ml eugenol-treated groups. Images A-D show the fat vacuoles in adipocytes whereas E-H show the calcium deposits in osteogenic cells. DMSO was enhanced the adipogenesis but decreased the osteogenesis. The effects of cinnamaldehyde and eugenol were in contrast with those of DMSO

Figure 5 shows that adipogenic and osteogenic differentiation have occurred in the presence of 0.01% DMSO, 2.5 µM/ml cinnamaldehyde or 0.1 µg/ml eugenol.

**Figure 6:**
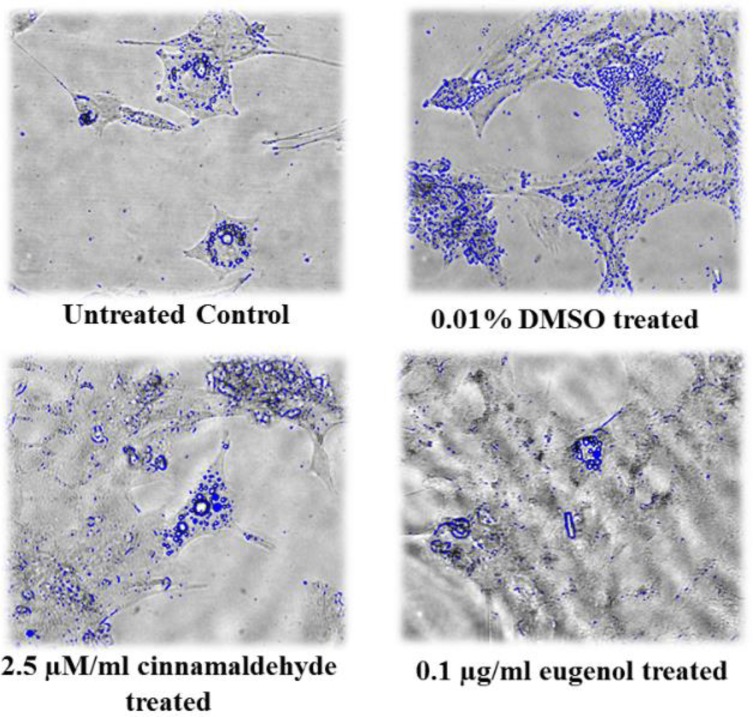
A sample of image analysis for counting the fat vacuoles in hASCs differentiated to adipocytes. Fat vacuoles were detected and counted using TotalLab TL120 software facilities. At least 5 images were analyzed for each studied group

**Figure 7:**
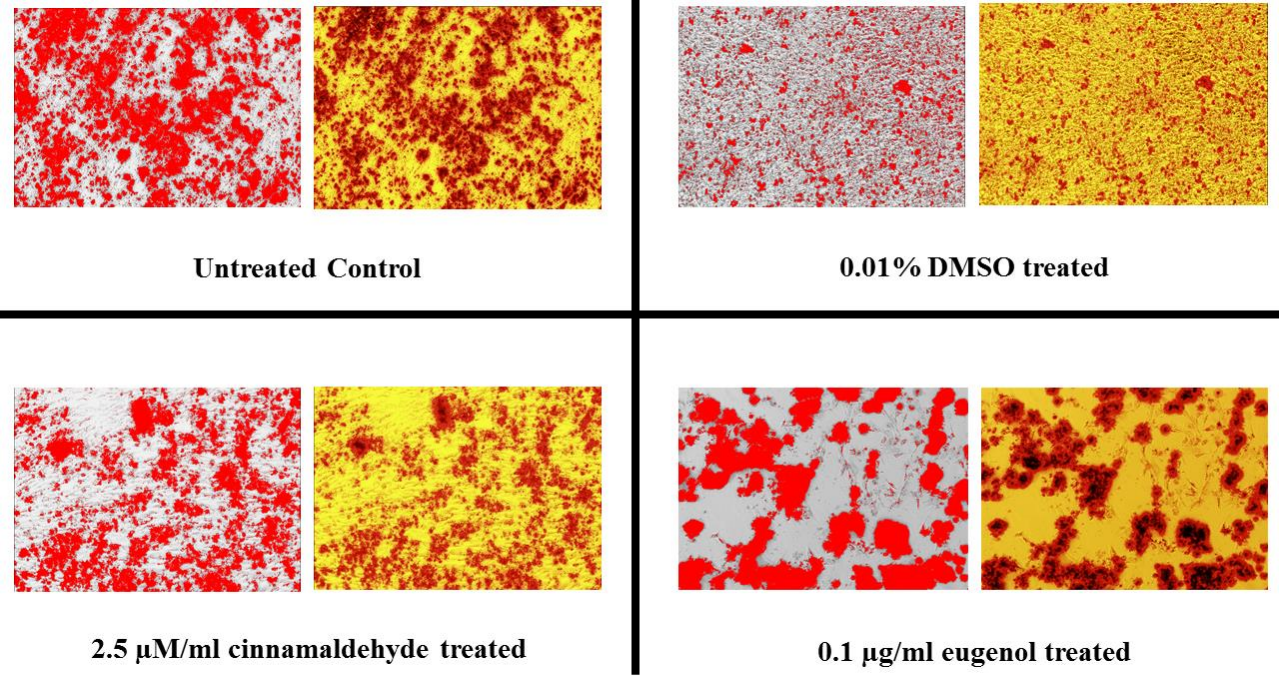
A sample of image analysis for calculation of calcium deposits in hASCs differentiated to osteocytes. Calcium deposits were detected as red color regions in each image and the percentage of the red color was estimated using ImageJ software facilities. At least 5 images were analyzed for each studied group

**Figure 8:**
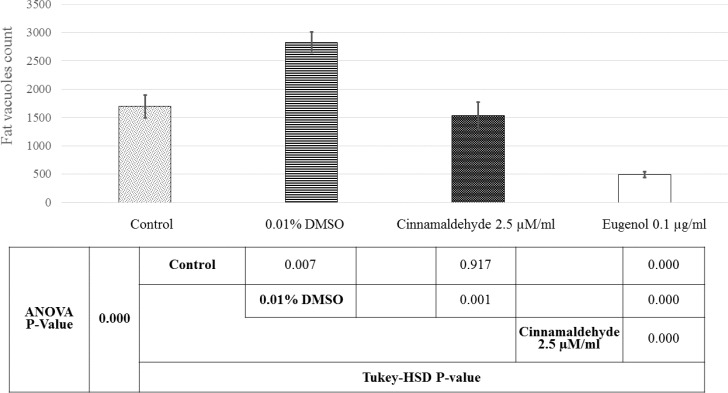
Comparative bar chart of fat vacuoles counts in the adipocyte differentiated hASCs. The fat vacuole counts were obtained by TotalLab TL120 software. Statistical comparisons are also shown below the histogram. Here, 0.01% DMSO concentration enhanced the adipogenesis whereas 0.1 µg/ml eugenol decreased it meaningfully. Although 0.01% DMSO was present in 2.5 µM/ml cinnamaldehyde and eugenol groups, both phytochemicals had a negative effect on the adipocyte differentiation of hASCs. The negative effect of 0.1 µg/ml eugenol on the adipocyte differentiation was more severe. Untreated cells were good representatives for basic condition and comparisons

**Figure 9:**
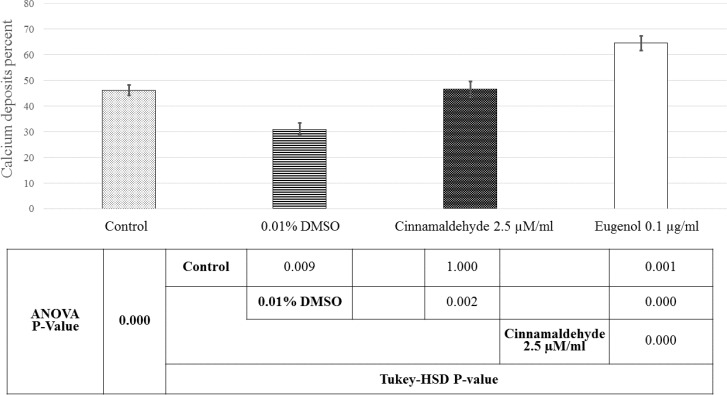
Comparative bar chart of calcium deposits percent in the osteocyte differentiated hASCs. The calcium deposits percent were obtained by ImageJ software. Statistical comparisons are also shown below the histogram. Here, 0.1 µg/ml eugenol enhanced the adipogenesis whereas 0.01% DMSO decreased it meaningfully. Although 0.01% DMSO was present in 2.5 µM/ml cinnamaldehyde and eugenol groups, both phytochemicals had a positive effect on the osteocyte differentiation of hASCs. The positive effect of 0.1 µg/ml eugenol on the osteocyte differentiation was more severe. Untreated cells were good representatives for basic condition and comparisons

Figures 6 and 7 show the samples of image analysis for fat vacuoles and calcium deposits using TotalLab TL120 and ImageJ softwares, respectively. Figures 8 and 9 represent bar charts and statistical comparisons among groups for adipogenesis and osteogenesis. These figures show detailed information and comparisons of results. Differentiation rates were significant between untreated and treated groups. The 0.01% DMSO in differentiation medium enhanced the adipogenesis but decreased the osteogenesis. Cinnamaldehyde and eugenol both decreased the adipogenesis but enhanced the osteogenesis. In comparison to the control group, the effect of cinnamaldehyde on the adipogenic and osteogenic differentiation was not meaningful. But it should be noted that 0.01% DMSO was also present in both the cinnamaldehyde-treated or eugenol-treated hASCs groups. Also, cinnamaldehyde group was compared with 0.01% DMSO group. By such comparison, the ultimate effect of 2.5 µM/ml cinnamaldehyde was a decrease in adipogenesis and an increase in osteogenesis. The effect of eugenol was clearly the decrease in the adipogenesis and the enhancement of osteogenesis rates which was significant as compared to all other treatment groups (CI=95%, p=0.000).

## Discussion

Table 1 shows that, based of toxicological tests, cinnamaldehyde and eugenol can be considered as non-toxic phytochemicals at certain concentrations. Also, according to the cheminformatics analysis, cinnamaldehyde and eugenol were expected to be non-toxic materials for hASCs, especially at concentrations about one thousand lower than the calculated LC_50_ or LD_50_. Overall, RO5 characteristics and cheminformatics evaluations suggested that: 1. Cinnamaldehyde and eugenol possess low or non-toxic characteristics ; 2. Cinnamaldehyde may be more fat soluble, weaker hydrogen donor or acceptor with lesser rotatable bonds, molecular weight and TPSA than eugenol; 3. Cinnamaldehyde is more permeable across living cell membranes and has low accumulation tendency in animal body than eugenol; 4. Both phytochemicals may be considered as developmental toxicants, but easily undergo degradation and metabolism. Tables 2 and 3 show the predicted end-products of cytochrome-P450 metabolism of cinnamaldehyde and eugenol, respectively. This implies that while examining cinnamaldehyde and eugenol *in vivo* or *in vitro*, their metabolism end-products are also important, considering their effective or toxic characteristics. For example, cytochrome-P450 of rat liver metabolizes eugenol to quinonemethide (Thompson et al., 1990 ▶), a class of reactive and electrophilic compounds with the ability of macromolecules alkylation induction (Auddy et al., 2003 ▶; Promega, 2012 ▶; Thompson et al., 1993 ▶).

In the current survey, the effect of three different concentrations of cinnamaldehyde and eugenol were tested on the cell viability and doubling time of hASCs. The MTS assay suggested that the applied concentrations could not limit the cell viability and metabolism, at least during the first 72 hr of treatment (p> 0.05 for 48 and 72 hr treatment). There were significant differences among groups for the first 24 hr. This finding is because of low accuracy of OD measurement for ODs below 0.3 (Promega, 2012); it should be noted that, in this work, all wells of culture plate had ODs<0.3 for 24 hr treatment (Figure 2).

Doubling time of hASCs was increased insignificantly in most cinnamaldehyde and eugenol-treated groups compared with control and DMSO-treated cells, other than 1µg/ml eugenol-treated hASCs. The increase in doubling times was concentration-dependent (Figure 4). This finding suggests that cinnamaldehyde and eugenol are toxic for hASCs at high concentrations. Further, the period of exposure to cinnamaldehyde and eugenol may also be critical for hASCs duplication but this was not clearly seen in the current work. Then, it is concluded that higher concentrations of investigated phytochemicals inhibit the cell growth rate of dividing cells. In this study, according to the doubling time of treated hASCs, the lowest concentrations of phytochemicals were selected for application in intervention studies; as they had better values than others.

In the current work, the viabilities of hASCs were not significantly changed among different studied groups, but doubling times were meaningfully different. Then, it is suggested that the doubling time assessment may be more sensitive and responsive than viability test to show the toxic effect of phytochemicals. In this study, three different concentrations of cinnamaldehyde were selected according to King and coworkers. They have shown that these concentrations could not change the HCT 116 cell line viability during three weeks of treatment (King et al., 2007 ▶). However, they have not reported the doubling time changes. But the present survey has shown that the doubling time is more important for dividing cells than metabolism and viability, because it is more sensitive and responsive to concentration changes. Further, the present work tested the hASCs without manipulations while King and coworkers used a Ras gene mutated cell line. Chen and colleagues have shown that different concentrations of eugenol change the half maximal inhibitory concentration (IC_50_) of 3T3 cell line and embryonic stem cells (Chen et al., 2010 ▶). The cheminformatics evaluations showed that the LC_50_ of eugenol for *Daphnia magna* equals 1.28 mg/l (µg/ml). Chen and colleagues have shown eugenol IC_50_ to be equal to 1.28 µg/ml in embryonic stem cells, experimentally. The highest concentration of eugenol used for hASCs treatment was 1 µg/ml which is close to cheminformatics estimation and mentioned experimental study on embryonic stem cells. These findings suggest that occasionally cheminformatics estimations may be closed to experimental data and could be predictive or confirmative, before or after interventional studies. However, experimental data did not confirm other cheminformatics estimations. In most cases, the cheminformatics results had overestimations in their calculations compared with experiments (compare Table 1 with data of Chen and coworkers study). Such conclusion suggests that cheminformatics and QSAR estimation software should strengthen their calculation algorithm and database.

As the best results of doubling time were obtained for 2.5 µM/ml cinnamaldehyde and 0.1 µg/ml eugenol-treated hASCs, in the current study, their effect on the adipogenesis and osteogenesis of hASCs were tested morphologically. Adipocyte differentiation was enhanced in 0.01% DMSO-treated hASCs compared to untreated controls, in adipogenic medium. This evidence suggests that DMSO, especially at low concentrations, not only is not toxic for hASCs but also may be helpful for their differentiation to adipocytes. Cinnamaldehyde and eugenol-treated hASCs had lower fat vacuoles than untreated or DMSO-treated ones, in the adipogenic medium. Also, these two phytochemicals may be beneficial for prevention of fat accumulation in the human body and reduction of stem cell differentiation to the adipose tissue. On the other hand, cinnamaldehyde and eugenol-treated hASCs had high percentages of calcium deposits, in the osteogenic medium, than DMSO-treated or untreated hASCs. These empirical evidence propose that DMSO may be toxic for osteogenesis whereas cinnamaldehyde and eugenol potentiate it. Moreover, cinnamaldehyde and eugenol maybe suggested as osteogenic nutritional supplements or bone forming agents via induction of hASCs differentiation to osteocytes. To our knowledge, mentioned findings about hASCs differentiation to adipocytes and osteocytes, are novel aspects of the current investigation.

However, cinnamaldehyde and eugenol were toxic at high concentrations for dividing cells. Therefore, they could be suggested as antineoplastic agents and may be useful in preventive medicine. Anticancer effect of cinnamaldehyde and eugenol were previously investigated in cell line models. Induction of apoptosis is the probable mechanism proposed for cinnamaldehyde and eugenol effect on the cancer cell lines (Jaganathan et al., 2011 ▶; Ka et al., 2003 ▶).

As the toxicological results of this report showed, cinnamaldehyde and eugenol, two important phytochemicals which are present in cinnamon bark, were of low toxicity and effective on induction or prevention of stem cells differentiation. So, they could be introduced as cell culture additives for regenerative medicine or tissue engineering. Genetic and epigenetic changes are other probable modes of action that could be evaluated in future investigations. Ultimately, these two phytochemicals need to be investigated regarding their usages and effects on human body. The present work is of worth because of its point of view about the effect of two important phytochemicals on non-manipulated human stem cells whereas many published studies have used manipulated cell lines.
